# Protein and amino acid digestibility: definitions and conventional oro-ileal determination in humans

**DOI:** 10.3389/fnut.2024.1407604

**Published:** 2024-06-20

**Authors:** Suzanne M. Hodgkinson

**Affiliations:** Riddet Institute, Massey University, Palmerston North, New Zealand

**Keywords:** protein, amino acids, amino acid digestibility, human, ileal digesta

## Abstract

When assessing protein quality, a correction needs to be made to take into consideration the availability of the amino acids. This correction is based on the digestibility of the amino acids. It is recommended to use ileal (end of small intestine) digestibility as opposed to faecal digestibility. A correction needs to be made for endogenous (gut sourced as opposed to diet sourced) amino acids to give true digestibility as opposed to apparent digestibility. Also, this correction should be made by correcting the amino acid composition for individual amino acid digestibilities as opposed to correcting all amino acids for nitrogen digestibility. Determination of true ileal amino acid digestibility requires the collection of ileal digesta. In the human there are two methods that can be used; naso-ileal intubation and using the ileostomy model. Both are discussed in detail and it is concluded that both are appropriate methods to collect ileal digesta.

## 1 Introduction

Typical diets contain a mixture of different protein sources which will vary in nutritional quality (amino acid composition and availability). The aspects that are evaluated when determining protein quality are the amino acid composition and availability of the amino acids. The relationship between the available amino acids and amino acid requirements is then determined. Amino acid digestibility, the disappearance of the amino acids from the gut following consumption of the protein source, is measured to determine amino acid availability. The first part of this work will define terms used in conjunction with amino acid digestibility.

## 2 Definitions

### 2.1 Faecal versus ileal digestibility

Amino acid or nitrogen digestibility were traditionally determined based on the difference between the amount of each amino acid or nitrogen consumed and the amount that appeared in the faeces. Faecal nitrogen digestibility is the basis for protein digestibility corrected amino acid score [PDCAAS; (1)] a method that is used commercially in countries such as the United States to evaluate protein quality.

The problem with the use of faecal amino acid or nitrogen digestibility to evaluate protein quality is that the large intestine contains large numbers of microbes which metabolise the amino acids as they pass through. In the pig it has been shown that over 80% of all of the amino acids present in the faeces are of microbial origin rather than dietary origin (2). Moreover, it is generally accepted that there is minimal if any absorption of intact amino acids in the large intestine. The latter has been shown by infusing a single dietary indispensable amino acid (lysine or methionine) into the colon of pigs that had received a diet that was first-limiting in the same amino acid(s) (lysine or methionine + cysteine). If the infused amino acid had been absorbed in nutritionally significant amounts, the nitrogen balance of the pigs would have improved. However, there was no change in the nitrogen balance of these pigs (3). Put together, this means that the absorption of amino acids in a form that can be used for protein metabolism finishes at the end of the small intestine; the terminal ileum. Faecal digestibility values will not represent the amount of amino acids digested and absorbed such that they partake in protein metabolism. Table 1 shows ileal (adult ileostomates) and faecal digestibility coefficients following the consumption of a meat-vegetable-cereal-dairy product-based diet and shows how the difference between ileal and faecal digestibility coefficients can be quite significant. For individual amino acids, differences of up to 0.15 (15% units) were reported. For accuracy, digestibility values must be determined at ileal level (thus giving ileal digestibility) to determine protein quality (1, 5).

**Table 1:** Mean ileal (determined in ileostomates) and faecal digestibility coefficients in adult human subjects consuming a meat/cereal/dairy – based diet^1^.

Amino acid	Ileal	Faecal	Statistical significance	Difference
Serine	0.87	0.92	*p* < 0.001	0.05
Threonine	0.85	0.89	*p* < 0.01	0.04
Glycine	0.72	0.87	P < 0.001	0.15
Methionine	0.93	0.83	P < 0.001	0.10
Tryptophan	0.77	0.83	*p* < 0.05	0.05

^1^Data from (4).

### 2.2 Apparent versus true digestibility

Calculating digestibility values based on the quantity of amino acids consumed in a food and the amount in digesta collected from the terminal ileum gives “apparent” ileal digestibility values. However, while digesta will contain amino acids of food origin, it also contains amino acids of endogenous origin. Endogenous secretions are those that originate from the gut as opposed to the food. Endogenous secretions include digestive enzymes secreted during the digestion process, mucous that lines the gut and enterocytes; the cells that line the gut and are regularly sloughed off and replaced. Serum albumin is also present in the endogenous secretions. Microbes, while not strictly endogenous, are also included in the endogenous category and their potential significance in terms of amino acid homeostasis is reviewed in Metges (6). The majority (around 70–80%) of the endogenous secretions are digested themselves and absorbed before the end of the small intestine (7). However, the remaining endogenous secretions will be present in the digesta.

To determine the amount of the eaten amino acids that are digested and absorbed, a correction needs to be made for the endogenous secretions, thus determining the amount of amino acids of dietary origin that are present in the digesta. When the digestibility is corrected for endogenous secretions (subtracting endogenous secretions from the total amino acid content in ileal digesta), “true” digestibility is determined. Standardized digestibility values are calculated in the same way as true digestibility (see below), thus this is an alternative term used by some research groups for true digestibility.

The equations to calculate apparent and true digestibility are given below.


$$\begin{array}{l} \mathrm{Apparent}\;\mathrm{digestibility}=\\ \frac{\mathrm{Dietary}\;\mathrm{amino}\;\mathrm{acids}-\mathrm{Amino}\;\mathrm{acids}\;\mathrm{in}\;\mathrm{ileal}\;\mathrm{digesta}}{\mathrm{Dietary}\;\mathrm{amino}\;\mathrm{acids}} \end{array}$$

$$\begin{array}{l} \mathrm{True}\;\mathrm{digestibility}=\\ \frac{\mathrm{Dietary}\;\mathrm{amino}\;\mathrm{acids}-\left(\mathrm{Amino}\;\mathrm{acids}\;\mathrm{in}\;\mathrm{ileal}\;\mathrm{digesta}-\mathrm{Endogenous}\;\mathrm{amino}\;\mathrm{acids}\right)}{\mathrm{Dietary}\;\mathrm{amino}\;\mathrm{acids}} \end{array}$$

The currently preferred method to quantify endogenous amino acids in ileal digesta, the [e.g., recommended by FAO Expert Working Groups; (8)] involves consuming a protein-free diet before collecting ileal digesta. When a protein-free diet is consumed, all of the amino acids in the digesta must be of endogenous origin. The value for endogenous secretions can be used to calculate true (or standardized) digestibility.

### 2.3 Correcting for nitrogen or individual amino acid digestibility

When determining protein quality, the correction from the total concentration of amino acids in a food/ingredient to the concentration of available amino acids can be based on nitrogen digestibility or individual amino acid digestibility values. If this correction is based on nitrogen digestibility, the total concentration of each amino acid is multiplied by the same value for digestibility; that for nitrogen. This method of calculation is used when PDCAAS is determined. The principle advantage of carrying out this correction based on nitrogen digestibility is the lower cost for the chemical analyses; it is a lot more economical to determine nitrogen in the samples than to determine the individual amino acids.

In samples both of foods/ingredients and digesta, not all of the nitrogen in a sample will be amino nitrogen. Thus nitrogen digestibility will include more than amino nitrogen. It is important to note that when individual amino acid digestibilities are examined, these can vary markedly in the same food/ingredient. This is especially the case when proteins with a lower average digestibility (60–75%) are considered, such as many cereals and legumes. Table 2 shows the true ileal digestibility coefficients for black beans [data from (9)]. Individual amino acid digestibility coefficients range from 0.302 for cysteine to 0.829 for reactive lysine. Using the nitrogen digestibility value (0.66) to correct the digestibility of all of the amino acids rather than individual amino acid digestibility values will result in inaccuracy in the digestibility data. Table 2 also shows the amino acid content and amount of digestible amino acids (9) calculated based on either the true ileal amino acid digestibility values of each individual amino acids or N digestibility for black beans. When the amount of true ileal digestible amino acids is calculated based on N digestibility, in this case the values are underestimated for all amino acids except cysteine (which is overestimated by 200%), with the underestimation ranging from 6.4% (threonine) to 20.3% for reactive lysine. In conclusion, to accurately determine protein quality, it is necessary to calculate the amount of available amino acids based on the true ileal digestibility of individual amino acids.

**Table 2:** Amino acid content, true ileal amino acid digestibility coefficient (TIAAD) and amount of digestible amino acids calculated based on the true ileal digestibility of individual amino acids or the digestibility of *N* for black beans^1^ determined in human ileostomates.

Amino acid	Amino acid content mg/g DM	TIAAD	Amount digestible AA (mg/g DM) based on	
			TIAAD	*N* digestibility
Threonine	10.6	0.705	7.5	7.0
Valine	12.4	0.743	9.2	8.2
Isoleucine	10.4	0.784	8.1	6.8
Leucine	18.6	0.797	14.9	12.3
Phenylalanine	13.5	0.809	10.9	8.9
Tyrosine	8.5	0.799	6.8	5.6
Histidine	6.7	0.736	4.9	4.4
Methionine	2.7	0.772	2.1	1.8
Cysteine	2.3	0.302	0.7	1.5
Reactive lysine	13.3	0.829	11.1	8.8
Tryptophan	3.0	0.727	2.2	2.0

^1^Data from (9).

## 3 Collection of ileal digesta from the human

The biggest complication with determining true ileal amino acid digestibility is that it requires the collection of digesta from the end of the small intestine, the terminal ileum, which is far from straightforward. Two methods that have been developed to collect ileal digesta from the human; naso-ileal intubation and with the participation of ileostomates. These are discussed below.

### 3.1 Naso-ileal intubation

Naso-ileal intubation is conducted with healthy adult participants. Under local anesthesia, a triple-lumen fine tube is inserted through the nose, down the back of the throat and into the esophagus. The tube then passes through the stomach and moves right to the end of the small intestine, the terminal ileum. One lumen of the tube is used to inflate a small balloon on the end of the tube to facilitate the movement of the tube through the small intestine via peristaltic movements. A non-absorbable marker (e.g., polyethylene glycol) is infused into the intestine through another lumen of the tube and digesta is collected via gentle aspiration through the third lumen, downstream from the site of marker infusion. The tube is radio-opaque and the correct positioning of the tube is checked via X-ray.

Once the tube is in position and after an overnight fast, a test meal with the only source of protein being the food/ingredient being tested (or a protein-free meal to determine endogenous amino acid losses) is consumed by the participant. For the following 8 h, the participant will only consume water, and digesta is gently and continuously aspirated through the tube to provide the ileal digesta sample. Calvez et al. (10) describes in detail the typical protocol for the use of naso-ileal intubation to determine true ileal amino acid digestibility.

The principle strength of the naso-ileal intubation method is that it allows ileal digesta to be collected from healthy “intact” participants. It does, however, have the limitation that it can be considered to be very invasive. Many participants are unable to tolerate the insertion and presence of the tube. Each participant can only partake in the testing of one food (or a protein-free meal). It is not an appropriate method for use in vulnerable groups such as children. It is an expensive technique and must be applied under hospital conditions. As the lumen of the sampling tube is small, if digesta contains many particles, these could clog the tube, which limits the foods that can be tested with this method.

One potential criticism of the method is whether the presence of the tube inside the gastrointestinal tract affects digestive function; such as gastric and/or intestinal transit time. Several studies have determined the effect of an intestinal tube on parameters such as gastric emptying. Some studies have reported a delayed gastric emptying (11–14) while Müller-Lissner et al. (15) reported little or no effect. Whether the presence of the tube affects parameters such as gastric emptying may not be important, however, as Gaudichon et al. (16) reported that amino acid absorption is not influenced by the transit rate of the food.

Overall, the naso-ileal intubation method appears to be a suitable method to collect ileal digesta from the healthy adult.

### 3.2 Human ileostomates

Human ileostomates are people that, due to medical conditions involving the large intestine, have the end of their small intestine surgically exteriorised via a stoma. Stoma bags are connected to the exterior of the stoma into which all of the digesta that passes through the small intestine are collected. When protein quality is evaluated with the ileostomy model, the participants consume a test meal following an overnight fast. The only source of protein in the test meal is the food/ingredient being tested (or a protein-free meal is consumed to determine endogenous amino acid losses). A fresh stoma bag is attached and all of the digesta that that enters the bag over the next 9 h is collected. While digesta are being collected, the participants can only consume water and sweetened drinks. Moughan et al. (17) describes a typical protocol for determining true ileal amino acid digestibility with the participation of ileostomates.

Working with ileostomates has the advantage that there is no limitation on the types (or particle size) of the foods that can be tested. Numerous protein sources/foods can be tested with each participant, although, due to the rigorous nature of the testing (no food can be consumed for 9 h after the test meal on study days), participants can reach study fatigue if there are too many study days over a short period of time.

The principal limitation involved in working with the ileostomy model is recruitment due to the low numbers of ileostomised people. Moreover, many ileostomised people have other health conditions or are prescribed medications that could affect digestive functions, so are not suitable for these studies. Nowadays it is common that after the ileostomy surgery and after the large intestine heals sufficiently, the ileostomy is reversed. This means that often there is only a short period of time between healing from the original surgery and having the surgery reversed, further complicating the recruitment of sufficient ileostomised participants for a study.

A potential concern with the use of ileostomised participants in nutritional studies is whether there is increased colonisation of the small intestine with microbes. Several studies have addressed this concern. Englyst and Cummings (18) evaluated polysaccharide digestion in ileostomised participants. The ileostomates consumed metronidazole, which inhibits the metabolism of anaerobic bacteria, with no differences found between before and after the consumption of metronidazole. Sandberg et al. (19) also concluded that there was little if any fermentation occurring in the small intestine of ileostomised participants. Fuller et al. (20) collected ileal digesta from ileostomates after they had consumed a protein-free diet. When antibiotics were administered and ileal digesta collected again, there was no difference in the concentration of amino acids compared with before the administration of antibiotics, also supporting that there is not an increased colonisation of the small intestine by microbes in ileostomised people.

### 3.3 Comparisons between ileostomised and intact people

Ileostomised participants have been used as models for the “intact” person, particularly to study digestion and absorption to the end of the small intestine both for protein (4, 19) and fiber (21). There is a considerable amount of evidence supporting the ileostomy model as a direct and quantitatively accurate model to evaluate nutrient digestibility in the upper gastrointestinal tract (18, 19, 21–27).

No differences have been found in the gastric or intestinal transit rate between ileostomates and “intact” people; the “head” of the meal has been shown to travel from the mouth to the terminal ileum in the same time in ileostomates as from the mouth to the caecum in “intact” humans (28).

A direct comparison of true ileal amino acid digestibility coefficients determined with naso-ileal intubation and the ileostomy model has been conducted. The true ileal amino acid digestibility of the protein sources zein (relatively low digestibility) and whey protein isolate (WPI, highly digestible) were determined using naso-ileal intubation [results reported in (10)] and with the ileostomy model [results reported in (9)] and the results were statistically compared. No statistically significant differences were determined (*p* > 0.05) between the methods for digestibility of either protein or for any amino acid. The calculated mean true ileal amino acid digestibility coefficients for zein were 0.63 and 0.60 and for WPI were 0.92 and 0.95 (naso-ileal intubation and ileostomy model, respectively). Thus the results for true ileal amino acid digestibility determined using the two methods do not differ. This information taken together supports ileostomised participants as being representative of the “intact” person to study the upper gastrointestinal tract.

The complications involved in collecting ileal digesta from humans mean that while they are useful methods for specific studies, these ileal digesta collection methods (naso-ileal intubation or with ileostomised participants) are not able to be used for routine analyses of multiple foods, for example to generate values required for DIAAS. This has led to the development of animal models, the use of which does have ethical implications. Direct comparisons between the growing ileal cannulated pig and ileostomised human have shown an excellent agreement of ileal amino acid digestibility values for a variety of different types of food (9). Thus the pig has been shown to be an excellent model for the human in terms of true ileal amino acid digestibility for when it is not possible or practical to collect digesta from the human.

## 4 Summary

Terms related to determining protein and amino acid digestibility are defined with recommendations made on which are considered to be the correct methods to use for protein quality determination, including the difference between faecal and ileal digestibility as well as apparent versus true digestibility. The correction for amino acid availability should be made with individual amino acid digestibility rather than correcting all amino acids for nitrogen digestibility.

There are two methods that can be used to collect digesta from the end of the small intestine; naso-ileal intubation and using the ileostomy model. Both are appropriate methods to collect ileal digesta and they are discussed in detail.

## Author contributions

SH: Writing – original draft, Writing – review & editing.
